# Selling my sheep to pay for medicines – household priorities and coping strategies in a setting without universal health coverage

**DOI:** 10.1186/s12913-018-2943-y

**Published:** 2018-03-02

**Authors:** Onarheim Kristine Husøy, Sisay Mitike Molla, Gizaw Muluken, Moland Karen Marie, Norheim Ole Frithof, Miljeteig Ingrid

**Affiliations:** 1 Department of Global Public Health and Primary Care, University of Bergen, P.O. Box 7804, 5020 Bergen, Norway; 2 School of Public Health, College of Health Sciences, Addis Ababa University, Addis Ababa, Ethiopia; 3 Centre for Intervention Science in Maternal and Child Health, University of Bergen, Bergen, Norway; 4 Harvard T.H. Chan School of Public Health, Harvard University, Boston, USA; 5 Department of Research and Development, Helse Bergen Health Trust, Bergen, Norway

**Keywords:** Universal health coverage, Catastrophic health expenditure, Poverty, Out-of-pocket expenses, Intra-household decision making, Resource allocation, Newborn health, Health care seeking, Ethiopia

## Abstract

**Background:**

The first month of life is the period with the highest risk of dying. Despite knowledge of effective interventions, newborn mortality is high and utilization of health care services remains low in Ethiopia. In settings without universal health coverage, the economy of a household is vulnerable to illness, and out-of-pocket payments may limit families’ opportunities to seek health care for newborns. In this paper we explore intra-household resource allocation, focusing on how families prioritize newborn health versus other household needs and their coping strategies for managing these priorities.

**Methods:**

A qualitative study was conducted in 2015 in Butajira, Ethiopia, comprising observation, semi-structured interviews, and focus group discussions with household members, health workers, and community members. Household members with hospitalized newborns or who had experienced neonatal death were primary informants.

**Results:**

In this predominantly rural and poor district, households struggled to pay out-of-pocket for services such as admission, diagnostics, drugs, and transportation. When newborns fell ill, families made hard choices balancing concerns for newborn health and other household needs. The ability to seek care, obtain services, and follow medical advice depended on the social and economic assets of the household. It was common to borrow money from friends and family, or even to sell a sheep or the harvest, if necessary. In managing household priorities and high costs, families waited before seeking health care, or used cheaper traditional medicines. For poor families with no money or opportunity to borrow, it became impossible to follow medical advice or even seek care in the first place. This had fatal health consequences for the sick newborns.

**Conclusions:**

While improving neonatal health is prioritized at policy level in Ethiopia, poor households with sick neonates may prioritize differently. With limited money at hand and high direct health care costs, families balanced conflicting concerns to newborn health and family welfare. We argue that families should not be left in situations where they have to choose between survival of the newborn and economic ruin. Protection against out-of-pocket spending is key as Ethiopia moves towards universal health coverage. A necessary step is to provide prioritized newborn health care services free of charge.

**Electronic supplementary material:**

The online version of this article (10.1186/s12913-018-2943-y) contains supplementary material, which is available to authorized users.

## Background

*I had nothing and I sold the only sheep I had to get treatment for my child. Before my child got sick, I was planning for the future; if the sheep gave birth I could send my children to school. So after I sold my sheep, my plan will fail… When the sheep is not there, what would I do in the future?*

(Focus group discussion, mother, rural Ethiopia)

In settings where user fees are high and patients and their families have to pay out-of-pocket for health care services, dilemmas arise regarding the use of available household resources: Should the family give priority to the needs of the sick patient, or the needs of the rest of the family?

Health care systems aim to improve the health and well-being of their populations. Closely tied to this objective is the need to avoid impoverishment when households use health care services. The interconnected relationship between health and financial risk protection has been framed as universal health coverage (UHC), which aims to ensure that everyone obtains the health care services they need without exposing them to financial hardship [1, 2]. The World Health Organization (WHO) recommends financial reforms of health care systems and incorporation of concerns for equity and fairness when countries move towards realization of UHC [1, 3]. UHC has affirmed its global importance as one of the Sustainable Development Goal (SDG) targets [2], and has received attention in national policy and planning. The promising momentum built around UHC needs to be translated into plans and implementation, and many countries have a long way to go. Despite the commitment to the ambitious goal of UHC, direct payments for using health care services, known as out-of-pockets (OOP) payments [4], contribute to half of total health expenditure in low income countries [5]. Without risk pooling through publicly financed health care systems, the economies of households remain vulnerable to illness. In health care systems that rely largely on OOP payments, health care costs can keep or push patients and their families into poverty [6, 7]. In response to high costs and limited health insurance options, households commonly rely on informal insurance through borrowing money and selling assets, known as financial *coping strategies* [7, 8]. In the short run, these strategies can work as buffers and make it possible for families to pay, but in the long run the effects can be damaging for the households and their resources [7, 9, 10]. When OOP payments exceed 40% of the household income after basic needs are met, they can be described as catastrophic [7]. A study from 2007 estimated that 150 million people globally suffer from catastrophic health spending every year [11], indicating the severity of the problem.

One country in which OOP payments are high is Ethiopia, where this study was conducted. Ethiopia is a low-income country in eastern Africa [12]. Remarkable development has been seen in an annual growth between 8 and 12% of gross domestic product (GDP) during the past five years [12], and through an increase of 9.1 years in life expectancy (from 56.1 to 65.2 years) between 2005 and 2015 [13]. While these trends are promising, one third of its population of almost 100 million lives below the poverty line [12].

The total health expenditures per GDP – both public and private – increased from 2.8% in 1995 to 4.7% in 2013 [5] (see Table 1), but are still far from the Abuja Declaration’s pledge to allocate 15% of the budget to health. The Ethiopian health care system is underfinanced in absolute numbers. In 2014, the health expenditure per capita was $27, which is substantially lower than what is recommended to uphold access to primary care services in low-income countries ($86) [14, 15]. Financing of health care services rely on OOP payments, and 34% of health care expenses are covered by households [15]. In this context, Ethiopia’s ambitious commitment to UHC seems warranted. The national health care system, primary care services and preventive care have been scaled-up through investments in health centers and community health workers (health extension workers). Further, community-based health insurance and social health insurance programs have been piloted in a selection of *woredas* (districts) [16, 17]. However, the increase in utilization of health care services is slow and the majority of the population remains uncovered by health insurance [18, 19].

**Table 1:** Economic development, poverty and health care financing

	Ethiopia	World
Annual GDP growth [12]	9.6%	2.6%
Gini index [16, 51]	33.2	70.5
Population living below the poverty line (<$1.90 a day (2011 PPP) [12]	33.5%	12.7%
Tax revenue (of GDP in 2011) [12]	9.2%	12.9%
OOP expenses for health care covered by households [15]	General: 34% Children 48%	
OOP per total health expenditure [5]	42.9%	20.6% (31.3% SSA)
Total health expenditure per GDP [5]		
1995	2.8%	6.0% (4.5% SSA)
2013	4.7%	7.1% (5.5% SSA)
Total health expenditure per capita 2014 (US$) [5]	27	1061

*GDP* gross domestic product, *PPP* purchasing power parity, *OOP* out-of-pocket, *SSA* sub-Saharan Africa

Every year, worldwide, 2.6 million newborns do not make it through their first 28 days of life [20, 21]. The major causes of neonatal deaths are preterm birth complications, intrapartum-related complications, and sepsis [22]. Despite the magnitude of the problem and knowledge of effective health care services for treating and preventing these conditions [23], the decline in mortality has been slower for newborns than for older children [20]. Although newborn mortality in Ethiopia declined from 47 to 29 newborn deaths per 1000 live births between 2005 and 2016 [18], neonatal disorders contributed to 14% of the burden of disease and 61,600 newborns deaths [24]. Newborn health has been prioritized in Ethiopian health plans, which has put emphasis on upgrading the quality of child and maternal health services and facilities [25]. Increasing utilization of effective interventions could avert newborn deaths [23], but coverage of essential newborn health care services remains low and unequally distributed. A newborn from a poor family, rural area or which the mother has low education is less likely to receive health services than other newborns (see Table 2) [18, 19]. Ethiopian households pay a larger share of health expenditures for children (48%) than for adults (34%) [15], pointing to the importance of studying family priorities. Globally, the literature on user-fees and utilization of newborn health care services is limited, and there are no studies from Ethiopia. The few studies that exist on OOP payments of hospital care for sick newborns find that costs are high, in particular for inpatient services and longer stays, where payments often exceed family income in low income families [26, 27]. Beyond the problem of health service delivery, structural barriers and social norms influence health care seeking. Earlier studies have shown a delay in recognition of personhood in Ethiopia, with implications for newborn illness and death [28].

**Table 2:** Use and inequality of health care services in Ethiopia

Background characteristics	Delivery in a health facility (%)	Postnatal checkup in the first two days after birth (%)
National average	26	17
Average by wealth quintile		
Highest quintile	65	41
Lowest quintile	12	9
Average by residence		
Urban	79	45
Rural	19	13
Average by mother’s education		
Mother more than secondary education	92	54
Mother no education	16	9

Data from the Ethiopian Demographic and Health Survey, 2016 [18]

In this setting without UHC, it becomes crucial to understand how families make choices about care seeking for newborns and health care spending. Decision making at the household level can be understood as intra-household resource allocation, where families make decisions about expenditures on health care, food, transportation, and other goods. Whereas the literature on UHC has identified dilemmas and trade-offs at the policy level [3], little is known about how households with limited resources prioritize between health and other needs. To understand more about intra-household resource allocation, family priorities regarding care seeking for newborns are of particular importance. Sick newborns require urgent care, and cannot make decisions themselves. In this study, we aim to explore intra-household resource allocation, focusing on how families prioritize newborn health and household needs in Ethiopia. Furthermore, we seek to explore coping strategies families use to manage these priorities.

## Methods

### Study setting

The study was conducted in the semi-urban town of Butajira and surrounding rural area. Butajira is situated in Gurage Zone three hours south of Addis Ababa. This area consists of farmland, in which the literacy levels are low and the poverty rate is high [29, 30]. The total fertility rate in the area is 5.3 [29]. The majority of the population are Muslims and Orthodox Christians.

Social and community-based health insurance schemes had not been implemented in Butajira at the time of data collection (October–November 2015). Butajira has one public and one private hospital to serve the population, with associated health centers in proximity of the town. The public hospital, as a part of the new three tier organization of the Ethiopian health care system, serves a population of 1–1.5 million. The hospital is open 24 h a day, and there is a health care professional on call, but services such as laboratory and radiology are fully open only during office hours on weekdays. The hospital commonly experienced shortages of drugs at the pharmacy and missing equipment. The pediatric unit consisted of 50 beds, and on weekdays one or two doctors were doing rounds at the ward.

In Butajira there is a health and demographic surveillance site: the Butajira Rural Health Program (BRHP). Established in 1987, BRHP registers and monitors births and deaths, and collects data on fertility and mortality in nine rural and urban kebeles (villages) [30, 31]. Information from BHRP facilitated data collection and identification of participants.

### Data collection and analysis

We chose a qualitative study design to capture the nuances and complexities in household decision making.

Data collection methods comprised in-depth interviews (IDIs) and focus-group discussions (FGDs), as well as observation and registration of costs of drugs, diagnostics, and other health care services. We conducted 41 IDIs with focus on direct experiences of newborn illness and 7 FGDs with emphasis on community perceptions (4–8 participants per FGD). There were three categories of participants with the aim of understanding family priorities from different perspectives: 1) household members that were experiencing newborn illness or had experienced newborn deaths the previous year (IDIs), 2) health workers involved in newborn or child health care (IDIs and FGDs), and 3) community members (FGDs) (see Table 3). The triangulation in methods and type of participants was intended to increase credibility of the study. Informants were recruited purposively by the primary investigator (PI; KHO) and research assistant (RA; MG) at the hospital, and community members and health workers were recruited through collaboration with the BRHP.

**Table 3:** Participants of in-depth interviews and focus group discussions

Type of participants	Recruitment of participants
Household members experiencing newborn illness or death (18–35 years)	
Mother or primary caretaker of sick newborn: 11 IDIs at hospital during illness, *9 follow-up IDIs* Mother or primary caretaker who faced newborn death: 5 urban IDIs, 5 rural IDIs	Sick newborn identified during hospital admission (> 1 day) by PI Recruited through Butajira Rural Health Program (BHRP)
1 IDI with key informant from health bureau	From health bureau
Health workers involved in newborn health care (20–35 years)	
3 IDIs with Medical Doctors 7 IDIs with nurses and midwifes 1 FGD with nurses and midwifes 1 FGD with health extension workers (HEWs)	From hospitals and health centers From hospital, health center and kebeles, HEWs through BHRP
Community members (20–73 years)	
1 FGD with women in reproductive age with child < 1 year (urban) 1 FGD with women in reproductive age with child < 1 year (rural) 1 FGD with husbands with wife with child < 1 year (rural) 1 FGD with grandmothers (rural) 1 FGD with religious leaders and elders (urban)	From communities in three selected kebeles, recruited through BHRP

The interviews focused on illness and care for the newborn, what costs the family had faced, and the impacts of seeking care for the newborn and the rest of the family. Attention was given to what was perceived as most important for the family when making decisions about care for the newborn.

By observation in the public hospital and in the participants’ homes, we aimed to gain additional understanding about family priorities and impacts of health-care seeking on the newborns and their families. Daily notes were taken and used for early analysis. Data about costs of health care services and related costs were collected in the public hospital, health center, and pharmacies, including costs of stay, drugs [32], equipment, procedures, diagnostics, and transportation. Interviews were conducted by the PI and RA in Amharic or English, depending on the informants’ preferences.

During data collection, the PI and RA discussed the topics that came up during daily debriefings. Interview guides and focus group guides (Additional file 1) were revised based on impressions and insights from data collection, and issues of particular interest were given further attention in subsequent interviews and in observations. The data were transcribed and translated from Amharic to English, and the written material was analyzed drawing upon qualitative content analysis [33]. Following preliminary analysis from data collection, the material was read in detail, organized, and coded by the PI, assisted by NVivo11 (https://www.qsrinternational.com/). Preliminary findings were discussed continuously by the team of authors during analysis and writing.

### Ethical considerations

The study was approved by the Institutional Review Board of the College of Health Sciences, Addis Ababa University, Ethiopia, and by the Regional Ethical Committee, Helse Vest, Norway. Informed consent (written or by fingerprint) was obtained from potential participants after they had received information about the study, the opportunity to take part, and indication that participation was voluntary. Participants received 100 birr (5 USD) to compensate for their time; this was a typical amount given to participants in previous studies in the area [34].

### Availability of data and materials

The data material cannot be made publicly available or available upon request in order to protect the identity of the participants of the study.

## Results

With limited money at hand, families had to strike between giving priority to long term economic security for the family on the one hand and taking the risk of spending scarce resources to save the life of the newborn on the other. The following section will describe how families faced tough choices between conflicting needs within the households. First, costs were perceived as a big burden, and families struggled to pay for health care and other expenses. Second, with limited money at hand, families faced hard choices, weighing concerns regarding survival of the newborn and damaging effects on the welfare of the family. Third, common coping strategies to pay for drugs, diagnostics, transportation, and other costs were to borrow from others or sell their assets. Fourth, when these coping strategies failed or were not feasible, poor families waited before seeking care or did not seek care at all, with dangerous consequences for the sick newborns.

**Fig. 1:**
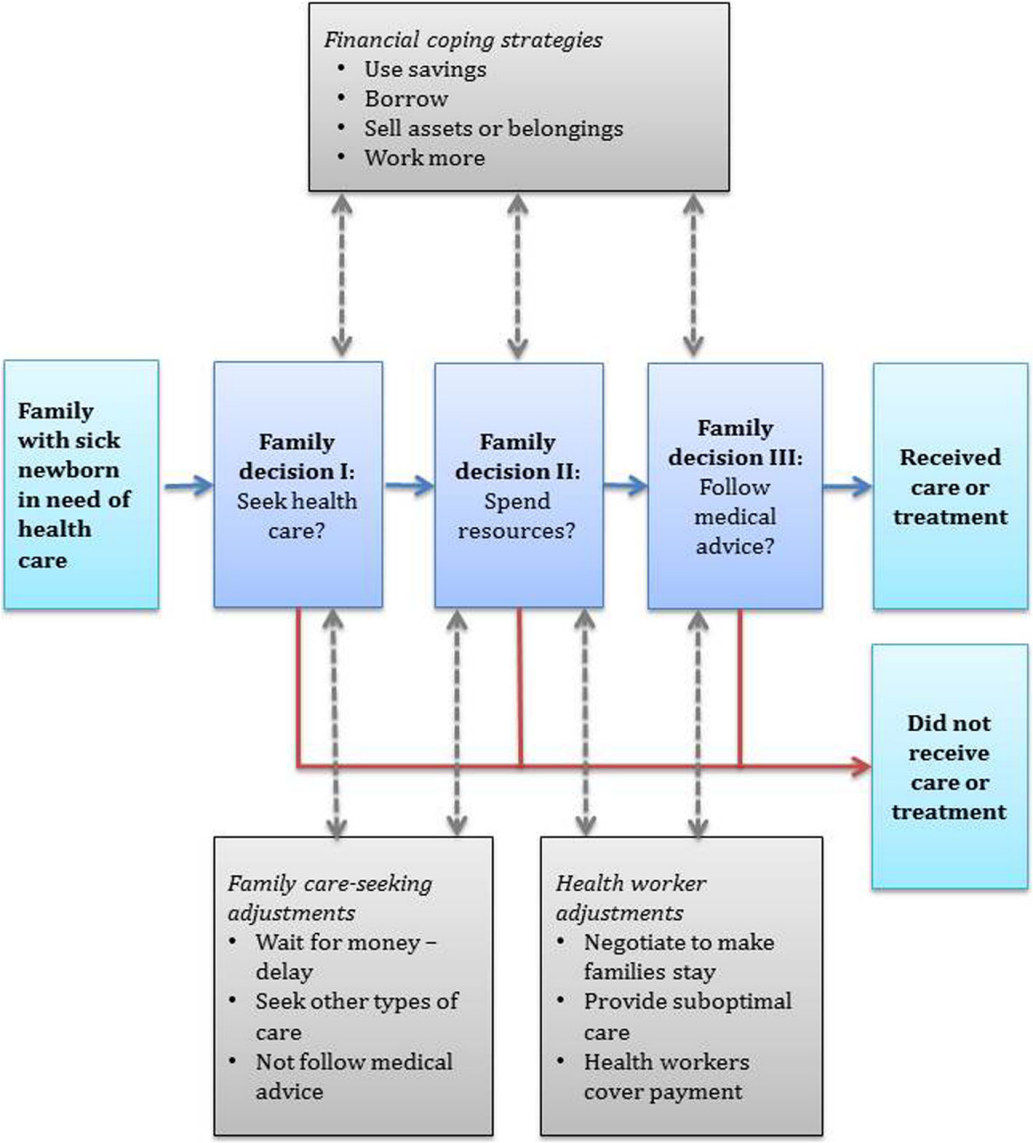
Family decisions on health care seeking for a sick newborn. Families made decisions about seeking health care (I), spending resources (II) and following medical advice (III). In these decision making processes families used financial coping strategies and made care-seeking adjustments. Health workers made adjustments to influence the families’ decisions and care seeking for sick newborns

Figure 1 illustrates decisions families made in the process of seeking care, spending resources, and following health care professionals’ advice. In making these decisions families used financial coping strategies and made care-seeking adjustments, and health workers made adjustments aiming to influence families’ decisions and care seeking.

### Facing high costs: ‘You have to pay for everything’

The costs of seeking care could be very high, and the costs troubled family members before, during, and after having used health care services. While delivery care was provided free of charge at the public hospital, families had to pay for services when the newborn was transferred to the neonatal unit in the same hospital. Community members noted that services were said to be provided for free, but in reality there were costs ‘for everything’.

*First you need money for card [the hospital’s individual patient record], then for laboratory, after that you need money to buy medicine and if the disease is severe, you need money for bed/admission, IV, injection. There is nothing free at the hospital. You have to buy everything. (FGD, mothers, rural area)*

Through observation at the hospital, we saw that for every new procedure or diagnostic test used, another amount was added to the bill. The families received information on how much they had to pay when they left the hospital. In addition to this bill, mothers or other household members were instructed by the doctors to buy drugs and other equipment, which could be bought at the hospital’s pharmacy or outside when the drugs were not available. Services provided at health centers or by health extension workers were less expensive or for free, but mothers described that drugs and other treatment were often not available there, which made it necessary to go to the hospital to get treatment. Health workers explained how the worry about costs made some fathers prefer treatment at lower-level facilities.

In addition to the direct expenses, families faced increased spending on food or other goods while away from home. Husbands and other family members went back and forth bringing food, gathering more money, or taking care of children who remained at home. Costs for transportation to health facilities by horse, public transport or ambulance could be high. While ambulances were most often free of charge for mothers, they were difficult to get hold of during night, and payments could be required for refueling after transportation to or from rural areas. Furthermore, long-distance travel to Addis Ababa for cases of referral could cost up to 1000 birr (45 USD) for the ambulance alone.

The families we met at the hospital and in follow-up interviews experienced high OOP payments, ranging from 600 (27USD) to 7000 birr (314 USD). These expenses included fees for health services, transportation, and other expenses related to seeking health care. Family and community members repeated how these expenses hampered care seeking, caused delay in seeking care, and burdened families with economic stress and worry.

### Making hard choices: ‘To treat the baby and let the family starve – Or not”

When newborns fell ill, families faced a series of decisions about seeking care, paying for care, and whether to follow medical advice (Fig. 1). Mothers and community members described the conflict between potential worsening of the baby’s health on the one hand, and risking unbearable costs and consequences for the family when taking the newborn to hospital on the other.

*Let us say a person has an ox with which he farms his land. If he sells this ox to be able to pay for treatment for his child, he will have nothing to fend his family with. In the end the family will be starved. They view this situation as a harmful thing. On the other hand, if he pays and treats his child, that is something you could call useful. (FGD, nurses and midwives, urban area)*

Mothers and fathers who had experienced newborn illness or death, health workers, and community members all emphasized the challenges of making these choices, but had somewhat diverging opinions on how these concerns should be weighted.

What mattered most to many mothers was saving the baby. When the baby was born too early or would not suck or in some other way needed care, mothers explained that they would seek care even if that meant leaving other children back home or selling their belongings.

*Human life and money are different things. Money is such a thing that we can get it if we work, but human life is irreversible if it’s once lost… It is understandable to think about the money, but whatever the fee is, there is nothing more precious than life. So we decided to bring him to health facility and spent all our money. We try to balance based on what we have, but we are worried about the money. (Mother experiencing newborn illness 6, urban area)*

Fathers and community members expressed worry regarding other family members and the consequences that seeking health care for the newborn would have on them. They stated that they could not be concerned only about the newborn, but had to think about the rest of the family as well. Health workers had experienced that when newborns and older children were admitted for longer stays, with high costs, but little improvement, fathers wanted to leave the hospital. As head of households, fathers were concerned about the family as a whole.

*They don’t want to spend a lot of money for one child when they have like seven or six back home. They are trying to find other ways to deal with the problems. (Health worker 12, urban area)*

Aiming to convince the families to stay, health workers explained that they negotiated with the families concerning health care for the babies. Yet, some family members would not *“sacrifice the whole family for only one child”*. At the same time, nurses and doctors noted the lack of options for poor families, and how these families could not prioritize concerns for the baby over the family economy.

The mothers’ presence at the hospital was difficult for those staying back home, as she was the primary care taker of the children and the one who managed cooking, feeding, and other needs. Mothers and family members at the hospital expressed worry about the rest of the household. Who would take care of the ones back home? Did the other children eat enough? If the parents were not at home or spent all the money on the sick newborn, the other children would suffer. One mother explained:

*If I go to the hospital with my child, there is no one who can properly give food for the others, there is no one to wash them or send them to school properly. They will not go to school and also there will be no one to buy them books. (FGD, mothers, rural area)*

Although the decisions and consequences for the newborns and the families varied, many families, and mothers in particular, found dealing with the burdens of illness and economic stress emotionally challenging, and experienced a sense of powerlessness. The ambiguous feelings related to the desire to take the sick baby to the hospital and the needs of the family as a whole caused worry during illness and admission. Some parents in urban areas were aware of medical treatment, and one father described the suffering when not managing to access adequate care in time. Their girl, who was born with fetal abnormalities, died while the family was mobilizing resources to go to Addis Ababa for referral.

*We were planning to take her there, and we tried, but we didn’t have enough money, and she died before I took her to Tikur Anbessa (tertiary hospital in Addis Ababa). I do cleaning in this city, which is how I live my life. If I stopped working to take care of her (the sick baby), my children would starve to death. I sold two hundred kg of maize, which was a reserve for future consumption of 1000 birr (45 USD), because there wasn’t any other option. I was trying to get 2000 birr (90 USD). Since I didn’t have enough money, she died before I took her there. I feel sad for not getting her treated; I would have felt better if she died while getting treatment at Tikur Anbessa. I swear to God, I get a headache whenever she crosses my mind; she didn’t get what she was supposed to get. The fact that I was unable to get her the treatment that she needed breaks my heart. (Father experiencing newborn death 2, urban area)*

### Finding a way to pay for care: ‘Selling my sheep’

After deciding to seek care for the sick newborns, families used different strategies to manage the high expenses they experienced. When a mother worried that her baby was sick, and wished to seek care, she needed money and had to mobilize resources. It was unusual to have cash available for care when someone fell sick, and the everyday economy depended on the families’ resources and exchanges of food, animals, land or other goods and assets. Many fathers were day laborers, where job opportunities and income could change from one day to the next. In these circumstances, families often had no money at hand nor were they prepared for the high expenses when someone fell ill.

*People living in the rural parts of the country do not save up money, which they could use as a health insurance. They don’t think they need money as a back-up if their child becomes ill. They often pawn their land or sell their herd to seek medical treatment for their sick children. Sometimes they ask us to be patient for the payments at the hospital when the money doesn’t arrive on time. (FGD, nurses and midwives, urban area)*

When both parents worked or the family had money available, their savings were the first option for covering the costs. However, few families had savings available, and the common strategy was to borrow from family members, friends or contacts in the neighborhood. To earn the money for repayment, the parents – often the fathers – had to work more or sell their harvest, animals or other assets. Staying at the hospital could be particularly damaging when the father lost income during the harvest season. One husband explained how they found money, and had to pay it back:

*Anyone who has the capacity will take money from home. A person who doesn’t have the money will borrow from close relatives or friends. In this way people will take their children to the health facility. After the child is cured the parents are obliged to pay the money they borrowed. If he has a tree that is ready he might sell some of it and pay his dept. The person may have a property like an ox, calf, sheep or goat. If the debt is small, he might sell the sheep and pay his debt. If the debt is large, he might sell two or three calves. If it is more than that or if his wife is ill, he has to sell the ox. (FGD, husbands, rural area)*

For some, paying back their debt was very difficult. There was less money for food or other resources, and one mother explained how her husband who was in debt had to leave his family for a while.

*I got treatment for my first child from the hospital and they charged us a lot of money. We did not have anything left after, and my husband was hiding. After a long time we were able to borrow money from a relative. Then we worked, and after some time we were able to pay the debt (FGD, mothers, rural area)*

At the hospital, mothers and families helped each other when they were out of money. They borrowed from each other, or gave money, drugs or food to mothers who did not have anything. Almost all mothers who had been in the hospital with their babies gave or received support from others in forms of money, drinks or medicines during their stay.

None of the families had made use of health insurance, nor did they mention it as a strategy to deal with high expenses. One family explained that they had heard about the introduction of a health insurance scheme. Health workers and religious leader described that the *kebele* (village) had a support system to aid poor people. Health workers explained that through this system, poor families could seek support to cover treatment costs. For care to be provided for free or at a reduced rate, a letter would have to be signed by leaders in the local kebele, based on a statement from one or more witnesses about the deprived economic status of the household. Husbands noted that this could be a time-consuming process at a time where urgent care was needed.

### When there are no assets to mobilize: ‘If the mother doesn’t have money, how can she take the child to the health facility?’

The poorest households or families with small networks could not rely on the previously described coping strategies. Neighbors and acquaintances were hesitant to lend them money, worrying that they would not be able to pay them back. Thus, poor families altered and adjusted their care seeking in accordance with the available resources. These strategies became visible through delays in health-care seeking, use of other types of care, or inability for families to follow the given medical advice.

*Only when the men have cattle, sheep and goat they will borrow – then they will be confident to receive their money by selling those assets. If someone doesn’t have any assets, no one is willing to give credit. Rather, they recommend different types of traditional medicine, saying it is better to give him some plant leaves, or explaining that it might be the devil and smoke some plastic sheets – (this advice is given) because of the fear that if I give him credit, he may not return (the money). But if the person has assets they are easily willing to give. (FGD, religious leaders, urban area)*

When families did not have money, they were advised by friends or neighbors to use traditional medicine, which was substantially cheaper, or that the illness was caused by evil spirits or spirit possessions. Health workers experienced that poor families came late, or with complicated cases, as they had waited a long time - even days - to get money. Mothers, community members and health workers noted that this deferral resulted in complications of illness.

There were some exceptions of mothers who left home with no money to seek care for their children. However, when they did so, they were aware of a way in which they planned to recover the costs later on. Families without money or support from others were not able to go to the hospital in the first place. They waited and hoped for the baby to get better, or were trying to get money to seek care. While waiting, some sick newborns did not make it to hospital, and did not survive. Further, families with some money faced similar challenges when they had borrowed or sold what they had, and struggled to follow the advice from the doctor and nurses about further treatment or referral. Newborns that needed care that was not available at the hospital were referred to higher level care, for tests at private health clinics or to hospitals in Addis Ababa. For referrals to Addis Ababa, the expected expenses for treatment and transportation were very high, and with limited resources families could not follow the recommendations. Health workers and household members described families who had been saving money, but in the meantime the condition of the baby worsened and became critical.

*The mother didn’t have the means to take her baby to Tikur Anbessa (central referral and teaching hospital), and she was forced to see her baby die at home. (FGD, nurses and midwives, urban area)*

Health care providers modified their recommendations in various ways if they recognized that costs were high and the fathers, or both parents, were hesitant or unable to pay. They tried to convince the family to seek care or to stay at the hospital, but if unsuccessful they suggested and provided some sort of treatment. Health workers repeatedly explained how they made use of leftover medications or tried to find alternative treatment options. These could include out-patient instead of in-patient services, fewer diagnostic tests or second-best medications. For very poor families or the rare cases of abandoned children, they even paid for drugs themselves.

## Discussion

### The health-welfare choices

This study illustrates families’ real-life dilemmas when newborns fall ill in a setting without UHC. In this deprived area, high health care costs and related expenses left families in situations where they had to choose between conflicting needs: Should the family sell their sheep to seek treatment for the baby? In other words, should individual health gains be compromised for concerns for family welfare? These hard choices between the newborn and the welfare of the family played out in every decision made, illustrated by three central decision steps for families (Fig. 1). First, should the family seek care? Second, should the family spend money on health care, and if so, how should they pay for services? Third, should they follow medical advice, and if so, how would they deal with the costs? The answers to these questions and the decisions made had implications for the whole family and their future. In intra-household resource allocation, families made compromises with effects on welfare and health outcomes. On welfare, families used financial coping strategies, such as borrowing or selling. On health, families adjusted the ways in which they sought health care. For the very poor living on the margin, the informal financial support mechanisms were not available, and through waiting for money and seeking other types of care, families made compromises affecting the health of the newborn.

### Methodological concerns

Some important methodological concerns should be noted. While this study focused on affordability, services must also be available, accessible, and appropriately and equitably delivered. Bottlenecks in the health workforce, financing, and service delivery create barriers to ensure essential maternal and newborn health care [35]. Families explained that health posts or health centers were not always effective or even open. The low quality of care at some facilities is another important reason as to why families do not seek health care [36]. Beyond these barriers, we believe that this in-depth study has extended our understanding through descriptions of the role that family priorities and coping strategies play in care seeking.

The key informants in the study were primary care takers that had experienced newborn illness or death, which brought unique and rich descriptions of intra-household resource allocation in these families. Community and health worker perspectives, observations, and knowledge of health service prices enabled triangulation of the sources of information. The results were presented and discussed with key stakeholders in Butajira (April 2017), which further strengthens the trustworthiness of the study. We chose deliberately to study families’ priorities and the trade-off between health and welfare in families from the perspective of newborn health. It should be noted that newborn deaths and stillbirths receive less attention than deaths of older children and adults [28], which might delay care seeking for newborns, as compared to adults. Further study on intra-household resource allocation between family members is needed [37].

We aimed to establish confidence in the discussion of sensitive issues through IDIs at the hospital and follow-up interviews at homes, and felt privileged but saddened to hear about families’ hard choices and dilemmas. The PI is a Norwegian medical doctor, and her understanding has shaped research questions, data collection, and analysis. Her earlier clinical experiences are mainly from settings where high quality health care services are provided for free. Her background and values may have made her particular attentive to the role of costs in care seeking, and potentially giving less emphasis to other important aspects of seeking health care for newborns. To better understand the local setting and perceptions, norms, and values, the data collection and analysis was conducted in close collaboration with MG and MM, who have extensive experience doing research in the area. While the ‘outsider’ view might have limited our understanding, it also made it possible to explore questions that an ‘insider’ could not have asked, such as *why* they would give priority to the health of the newborn or to the welfare of the household.

### Household priorities in poverty settings

Banerjee and Duflo’s important work on the complex economic lives of the poor describes how people living in poverty have higher risks of unfortunate events, and how changes in income or high expenses have relatively larger impacts on their already limited expenditures [38]. High health care expenses can be a burden, or even a catastrophe, for patients and their families, and can lead to impoverishment. In Butajira, as seen elsewhere [7, 9–11], financial coping strategies, such as borrowing money and selling assets, were used as a source of informal insurance that enabled families to seek care. However, the poor cannot rely on the same coping mechanisms, and are not protected against catastrophic health expenditure through these informal community-based strategies [38–40]. This study described how families experiencing illness in a setting without social and community-based health insurance faced large economic stress and high OOP expenses. This seemed to be in particular damaging for the poor, who did not have access to financial coping strategies and made adjustments when choosing if and when to seek care.

The circumstances in Butajira – with high poverty, low literacy, and varying quality and availability of care – shaped families’ abilities to make choices. Despite aspirations and expressed wishes to seek care, the unbearable costs of care and concern for the family’s future represented a persistent challenge. From a societal perspective, we argue that these families and patients – and in particular poor families – did not have the opportunity to seek health care and be healthy. Amartya Sen, in his capabilities approach, argues that policies should be judged based on the freedoms or capabilities people have to ‘lead the kind of lives they value – and have reason to value’ [41]. Therefore, when studying household priorities, we must also look at their capacity to make free choices [41, 42]. Families’ decisions that directly or indirectly delayed care seeking in Butajira can be understood as a choice between family welfare and newborn health, where poor families gave priority to family welfare over newborn health. Others could claim that the repeated efforts by family members to seek care, despite harmful consequences, imply that they might have chosen to seek care if they had money, but were limited by actual opportunities. We argue that the ability to pay was decisive for the actual opportunity to seek health care, and that families, and in particular the newborns, do not have the capability to lead the kinds of lives we assume they would have reason to value. Building on Sen’s approach we argue that, from a societal perspective, this injustice must be addressed by policies that secure families actual opportunities to seeking- and paying for care.

### UHC, financial risk protection, and newborn health care services in Ethiopia

Child and maternal health services are supposed to be provided free of charge at the health center level, but families struggled with high expenses for newborn care at the hospital. Formal health insurance was not available in Butajira, and the ability to seek care, pay, and follow medical advice depended on the economic situation of the household. This finding illustrates the reality of patients and their families in a health care system that relies on OOP spending [5], and is in line with other studies describing the large burden of OOP costs in Ethiopia [43, 44]. In realization of UHC, WHO recommends that national policy makers set priorities regarding which services to cover, who should be covered, and how to proceed from OOP spending towards prepayment systems [1, 3]. OOP expenses cannot be eliminated for all services at once, and WHO recommends eliminating co-payments on high-priority services [3], which can be promoted through prepayment and risk pooling by using health insurance schemes and reimbursement systems. The burden of newborn mortality remains high, and if newborn health care is a continued priority towards UHC in Ethiopia [25], efforts to reduce or eliminate co-payments of these and other priority services is necessary.

Although there are limitations in the quality of published studies, removal of user fees has generally been associated with increased utilization of health care services [45, 46]. However, studies have shown disruptive effects when user-fee removal is implemented in unstable health systems [47], and the varying impact on health outcomes highlights the importance of quality of care [48]. The removal of user fees may have positive effects on welfare, seen in the reduction of OOP expenses and catastrophic health expenditure [40, 49]. One year after abolishing user fees for children in Burkina Faso, the risk of households experiencing expenses at health facilities was reduced by two-thirds [49]. In Ethiopia, a pilot community-based health insurance scheme was introduced in 13 *woredas* (districts) in 2011. The pilot found increases in outpatient health care utilization and reductions in the need to borrow [16, 17]. Current efforts under the Ethiopian Health Insurance Agency to introduce voluntary community-based health insurance to individuals and families in the informal sector, and compulsory social health insurance through the formal sector, are promising steps in accelerating progress towards UHC [50]. However, these initiatives are not scaled-up nationwide yet, leaving 95% of the population without health insurance [18]. Further, coverage of high priority newborn health services remains low, as seen in slow increase of postnatal care from 7% in 2005 to 17% in 2016 [18, 19]. With this as a backdrop, our findings illustrates that the reality is far from the ambitious goal of UHC, and efforts must be accelerated to realize UHC.

## Conclusions

When countries move towards UHC, financial risk protection from catastrophic spending on health care is essential for improved health and in avoiding harmful effects on family welfare. This study describes how families in Butajira, without risk pooling and prepayment systems in place, faced hard choices when their newborns fell ill. In intra-households resource allocation families balanced conflicting concerns to newborn health and family welfare. To manage and cope with the high costs, families borrowed money, sold assets and adjusted their care seeking. From a societal perspective, we argue that families should not be left in situations where they have to choose between health and welfare, between the survival of the newborn and selling their sheep. Steps towards UHC and efforts to secure financial risk protection through implementation of community-based health insurance and social health insurance are promising. Prioritized essential child-health services, including neonatal health care services, should be delivered free of charge to protect against financial catastrophe and to improve newborn survival.

## Additional file

Additional file 1: Interview and focus group guides. (PDF 299 kb)

## Abbreviations

FGD: focus-group discussions
GDP: gross domestic product
IDI: in-depth interviews
OOP: out-of-pocket
PI: primary investigator
PPP: purchasing power parity
RA: research assistant
SDG: sustainable development goal
SSA: sub-Saharan Africa
UHC: universal health coverage
WHO: World Health Organization
