# Supplementary material for: Selling my sheep to pay for medicines – household priorities and coping strategies in a setting without universal health coverage
Source: BMC Health Serv Res. 2018 Mar 2;18:153. doi: 10.1186/s12913-018-2943-y (PMC5833112; doi:10.1186/s12913-018-2943-y)
Supplement: Supplementary file 1 — Interview and focus group guides. (PDF 299 kb) [file 12913_2018_2943_MOESM1_ESM.pdf]

INTERVIEW AND FOCUS GROUP GUIDES

Interview guide: Family members at health facility

Site:

Date and time:

Name of interviewer:

Introduction

Thank you for agreeing to speak with us today. My name is ___________ and my colleague's name is ________. We are researchers at the School of Public Health, Addis Ababa University, Ethiopia and the Department of Global Public Health, University of Bergen, Norway. We are doing a study to learn more about care of sick babies and the use of health care services. We hope that this study will give important knowledge to improve health care services to newborns.

Since you recently experienced that your baby was ill, we would like to invite you to participate in this study. If you are willing to participate in the study we would like to interview you today about your experiences related to your baby's illness (at discharge from hospital) and then at home after two weeks.

Procedure

We are interested in your thoughts and experiences, and there are no right or wrong answers. Before we begin, I would like to read aloud the informed consent form.

[Interviewer reads the informed consent and asks the participant whether he/she has any questions. The participant will sign the form or withdraw from the study]

As I mentioned [in the informed consent], I would like to use a tape recorder to record our conversation. I would like to emphasise again that the information you provide, will be confidential. Any information we use from your interview will be combined with other information and it will not be possible to identify what you said. Is this acceptable?

Do you have any questions before we start the interview?

Before we start with some questions, can you tell us about yourself?
Potential areas to be included:

- Interviewee : Age, Gender, Work, Religion
- Size of household, Marital status
- Number of pregnancies, Number of live children
- Wealth measure: Parabol? Radio? Roofs? Smart telephones?
- Gender, Age and Delivery place of newborn that has been ill

Question 1: I'm very sorry to hear that your baby has been sick. Can you please tell me what happened from the time your child was born and up to now?
Probing:

- How did you come to understand that your baby was sick? (Own observation, information from doctor, child not sucking well, child not crying, febrile, breathing problem, etc.)
- What happened next?
- Did you have any thoughts about what kind of sickness the baby had?

Question 2: What did you do when you realized that she/he was sick?
Probing:

- Treated him/her at home?
- Did you seek help? Where? (Relative? Religious leader? Holy water? Health extension worker? Health development army? Health center?)
- What were the reasons for seeking care…?

Question 3: What advice or treatment was given from the persons you sought help from?
Probing:

- What did you think of the advice you got?
- What did you think of the advice you got here (at health facility)?
- Did you follow the advice? Why/why not?

Question 4: How did you decide to seek care at this health facility?
Probing:

- Advice from HEWs, other health care workers?
- Who were involved in making the decision? Mother, father, older siblings, mother in law, grandmothers, others?
- If there was disagreement, what was it about? How did you agree?

Question 5: Do you have any earlier experiences with seeking care for sick babies?

- In your family? Among you friends?
- How was this of relevance when your newborn got sick this time?

Question 6: What was important when you decided to [to seek care at health facility/stay at home/go to traditional practitioner]?
Probing:

- Money, transport, food while in hospital?
- Hope of survival, lack of confidence in survival, other priorities in the household?

Question 7: How did potential costs of care for treatment for the sick baby influence your decision to seek help?
Probing:

- How much were the costs? How did you manage to mobilize/get the money needed?
- If another family member had fallen sick and you had to choose, would you bring the baby or {other family member} to the hospital? Why/why not?
    - Imagine that your mother has stomach pain. She has experienced stomach pain from time to time the last months, but it has now gotten worse and she cannot help out in the house anymore. At the same time, your baby is not sucking well

and is very sleepy. What do you do? What kind of expectations will other people have on this? Should you take your mother to the hospital? The baby?

Question 8: Do you have any earlier experiences of high costs of treatment when family members have fallen sick?
Probing:
- What happened? Loan? Selling? Borrowing from relatives? Health insurance?
- How do you think these experiences may have influenced your decision this time?

Question 9: How has the illness of the baby influenced the everyday life for you and the rest of the family?
Probing:
- Did you have any additional expenses?
- E.g. missing school, missing to work, use of family resources, food, time spent on other activities, other children, savings?
- Did you have to spend resources differently; Less spending on food/education/transportation? Selling something?

I have now asked you several questions about illness of your baby. I have a general question which you might have some thoughts on:

Question 10: Some say that newborns are less important than adults, while others say that newborns are more important than adults: How is illness of newborns perceived versus illness of adults in your community?
Probing:
- How does your opinion differ from this?

Question 11: Do you have anything that you would like to add that we have not talked about?

Thank you for your participation and time.

Interview guide: Family members – follow-up interview

Site:

Date and time:

Date and time of interview I (at health facility):

Name of interviewer:

Introduction

Thank you for agreeing to speak with us again today. We met {two} weeks ago, but I would like to introduce myself and the project again. My name is ___________ and my colleague's name is ________. We are researchers at the School of Public Health, Addis Ababa University, Ethiopia and the Department of Global Public Health, University of Bergen, Norway. We are doing a study to learn more about care of sick babies and the use of health care services. We hope that this study will give important knowledge to improve health care services to babies.

Today, we would like to conduct a follow-up interview after we met in the hospital about {two} weeks ago, as we also are interested what happened after you left the hospital.

Procedure

We are interested in your thoughts and experiences, and there are no right or wrong answers. Before we begin, I would like to read aloud the informed consent form.

[Interviewer reads the informed consent and asks the participant whether he/she has any questions. The participant will sign the form or withdraw from the study]

As I mentioned [in the informed consent], I would like to use a tape recorder to record our conversation. I would like to emphasise again that the information you provide, will be confidential. Any information we use from your interview will be combined with other information and it will not be possible to identify what you said. Is this acceptable?

Do you have any questions before we start the interview?

Question 1: I was very concerned that your child was sick when we met two weeks ago. Can you please tell me how you and the baby are doing?
Probing:

- What happened from the day we spoke (at discharge) and up to now? How was the travel home? How has it been to staying at home?
- Did the condition of the baby improve or worsen after arriving at home? How did you understand that he/she was doing better/worse (own observation, information from health care worker, baby not sucking, baby not crying, febrile, breathing problem)? What did you do? Consulted other practitioners, relatives, neighbors, took the baby back to health facility?)

Question 2: What did they do in hospital/health center [to make him/her better?]
Probing:

- What did you think of this?
- How long did you stay in the health facility?
- How was it for you to be admitted to the hospital/health center?

Question 3: What advice or treatment was given from the health personnel you got help from?
Probing:
- What did you think of the advice you got?
- Did you trust the advice you got? From the doctor? From the nurse? Others at the hospital/health center?
- Did you seek help from other? Relatives? Religious leader? Health extension worker? Health development army? Health center?

Question 4: What was important when you made the decision to follow the recommendations from the doctor/not follow the recommendations?
Probing:
- If there was disagreement, what was it about?
- How did money, transport, food while in hospital play a role?

Question 5: Who were involved in making the decision to stay at/leave the hospital?
Probing:
- What were the roles of the mother, father, older siblings, mother in law, grandmothers, others?

Question 6: What costs did you experience related to the stay in the health facility?
Probing:
- What consequences did these costs have for your family budget after discharge?
- *Did these costs influence how you spend your resources (savings, valuables, etc)?* Less spending on food/education/transportation? Did you have to sell something?
- Now looking back, how do you consider the decision to spend the money on health care for the baby?

Question 7: How has the illness of the baby influenced the everyday life of the rest of the family after you left the hospital?
Probing:
- What were the consequences for the other children in the family? Use of family resources, food, time spent on other activities, savings of the family?

Question 8: Last time, I brought up a statement, I would like to hear your thoughts on again: Some say that newborns are less important than adults, while others say that newborns are more important than adults. Given your experience with your sick baby, what do you think of the statement?
Probing:
- What is the understanding in the community about this? In your family?
- How has the experience of having a sick baby shaped your thinking about this?

Question 9: Do you have anything to add that you would like to add that we have not talked about?

Thank you for your participation and time.

Interview guide: Family members experiencing newborn death

Site:
Date and time:
Name of interviewer:

Introduction

Thank you for agreeing to speak with us today. My name is ___________ and my colleague's name is ________. We are researchers at the School of Public Health, Addis Ababa University, Ethiopia and the Department of Global Public Health, University of Bergen, Norway. We are doing a study to learn more about care of sick babies and the use of health care services.

We would like to invite you to participate in this study as we have understood that you recently experienced that your baby got sick, and passed away. We are very sad to hear about this. We hope that this study will give important knowledge about health care for ill babies, and that it can help preventing deaths for other babies that fall sick in the future.

Procedure

We are interested in your thoughts and experiences, and there are no right or wrong answers. We are very sorry to hear that you baby passed away. We understand that this must be a very difficult time for you. We appreciate that you agree to talk to us, but would like to emphasize that you at any time can stop this interview.

Before we begin, I would like to read aloud the informed consent form.

[Interviewer reads the informed consent and asks the participant whether he/she has any questions. The participant will sign the form or withdraw from the study]

As I mentioned [in the informed consent], I would like to use a tape recorder to record our conversation. I would like to emphasise again that the information you provide, will be confidential. Any information we use from your interview will be combined with other information and it will not be possible to identify you. Is this acceptable?

Do you have any questions before we start the interview?

Before we start with some questions, can you tell us about yourself?
Potential areas to be included:

- Interviewee : Age, Gender, Work, Religion
- Size of household, Marital status
- Number of pregnancies, Number of live children
- Wealth measure: Parabol? Radio? Roofs? Smart telephones?
- Delivery place of baby
- Gender of baby
- Age of baby at illness, Age of baby at death

- Question 1: Can you please tell me what happened from the time your baby was born and until he/she became sick and died?
  Probing:
    - How did you come to understand that your baby was sick? (own observation, information from doctor, child not sucking well, child not crying, etc)
    - What happened next?
    - Did you have any thoughts about what kind of sickness the baby had?

  Question 2: What did you do when you realized that she/he was sick?
  Probing:
    - Treating him/her at home?
    - Did you seek help? Where? Traditional healers? Relative? Religious leader? Health extension worker? Health development army? Health center?
    - Why did you seek care…?

  Question 3: What advice or treatment was given from the persons you got help from?
  Probing:
    - What did you think of the advice you got?
    - Did you follow the advice? Why/why not?

  Question 4: How did you decide to seek care at health facility /stay at home/go to traditional practitioner?
  Probing:
    - Who were involved in making the decision? Mother, father, older siblings, mother in law, grandmothers, others?
    - If there was disagreement, what was it about? Did you agree? How did you agree?

  Question 5: What were the reasons for you to seek care/stay at home/go to traditional practitioner?
  Probing:
    - Why?
    - Belief that the outcome was in the hands of God, costs of treatment, transport or food while in hospital?
    - Hope of survival, lack of confidence in survival, other priorities in the household?

  Question 6: Do you have any earlier experiences with seeking care for sick babies?
    - If so, to what extent did your experiences help you when your baby got sick this time?

  Question 7: How did costs of care influence your decision to seek care when your baby fell sick?
  Probing:
    - How much did you pay for…?
    - Which other costs did you have?
    - What costs have you had after the baby died? Funeral? Other costs?

  Question 8: Do you have any earlier experiences of high costs of care when a family member has fallen sick?
  Probing:
    - What happened? Loan? Borrowing from relatives? Health insurance?

- How do you think these experiences may have influenced your/your family's decision this time?

Question 9: What was in your greatest challenge in connection with the death of your baby?

- Personal loss, reputation as a mother, blame from family members, rumours, loss of self-esteem?
- Expenses? Funeral costs?
- How are you coping with this now?

Question 10: How has the illness and later death of the baby influenced the everyday life for you and the rest of the family?

Probing:

- Did you have any additional expenses?
- What were the impacts on other family members, e.g. going to school, going to work, use of family resources, food, time spent on other activities, other children, savings?
- Did you have to spend resources differently; Less spending on food, education, transportation? Funeral costs? Are you working as you used to do before?

I have now asked you several questions about illness of your child. I have a general question which you might have some thoughts on:

Question 11: Some say that newborns are less important than adults, while others say that newborns are more important than adults: How is illness of newborns perceived versus illness of adults in your community?

Probing:

- How does your opinion differ from this?

Question 12: Do you have anything you would like to add to what we have talked about?

Thank you for your participation and time.

Interview guide: Health care workers

Site:
Date and time:
Name of interviewer:

Thank you for agreeing to speak with us today. My name is ___________ and my colleague's name is ________ We are researchers at the School of Public Health, Addis Ababa University, Ethiopia and the Department of Global Public Health, University of Bergen, Norway. We are doing a study to learn more about care of sick babies and the use of health care services. We also seek to understand the social and economic consequences for families with sick babies. We hope that this study will give important knowledge to improve health care services to newborns.

We would like to invite you to participate in this study and will ask your permission to interview you as a health professional with experience in caring for sick newborns.

Procedure

We are interested in your thoughts and experiences, and there are no right or wrong answers. Before we begin, I would like to read aloud the informed consent form.

[Interviewer reads the informed consent and asks the participant whether he/she has any questions. The participant will sign the form or withdraw from the study]

As I mentioned [in the informed consent], I would like to use a tape recorder to record our conversation. I would like to emphasise again that the information you provide, will be kept confidential. Any information we use from your interview will be combined with other information and it will not be possible to identify you. Is this acceptable?

Do you have any questions before we start the interview?

Before we start with some questions, can you tell us about yourself?
Potential areas to be included:

- Interviewee : Age, Gender,
- Profession, Role at health facility
- Years of experience at health facility, Year of graduation
- Marital status, Number of children, Size of household,
- Religion

Question 1: We have previously talked about some cases of newborn illness in the ward, and we would like to discuss with you the challenges you meet in caring for sick newborns in the hospital/health centers. Can you tell me about the challenges you have to handle when you are treating sick newborns?

Probing:

- What kind of health problems among the newborns do you see?
- Can you tell me about a sick newborn you met lately?

Question 2: Based on your experience, when and possibly for what conditions do the families seek care at health facilities if a newborn is ill? When do the families *not* seek care?

Probing:
- Why do you think it is like this?
- Do they seek help from others? Traditional healers? Relatives? Religious leader? Health extension worker? Health development army? Why?

Question 3: How do you think earlier experiences with health care seeking (for sick newborns, other family members) influence their care seeking when their newborn is ill?
Probing:
- Why is earlier experience(s) relevant to the families?
- Can you given an example?

Question 4: How do families adhere to medical advice that you give when the baby is ill?
Probing:
- Can you give an example where they did not follow you advice? E.g. not taking treatment, leaving the hospital against your advice, etc.?
- Can you give an example where they did not follow you advice? Why do you think they (this family) followed your advice?
- Why do they (not) adhere?

Question 5: How would you as a health care professional respond to a family that does not follow the advice you give them?
Probing:
- E.g. leaving the hospital against you advice, not taking treatment, etc?
    - Do you play a role in protecting the interests of the baby? The interests of other family members?

Question 6: Have you ever experienced that a baby has been left behind in the hospital? What did you do?
Probing:
- Why do you think the family / parents left the baby at the hospital/health center?
- What happens to these babies?
- Who are responsible for taking care of these babies? At the hospital? In the community?
- In the case you just described… Did you recognize that they were leaving the hospital? If so, how did you understand that they were leaving?

We will now discuss difficult decisions the family might have to make when a newborn is ill.
Question 7: Based on your experiences from the hospital/health center, which concerns are (most) important for the family when a newborn fall ill?
Probing:
- Is the newborn the key priority? Why/why not?
- Can you give an example from the ward?
- What if one of the older brothers, the mother, the father, or the grandmother (father's mother) also has fallen sick. If they have to choose, do you think they treat the baby or the others?
- Why? How are these concerns balanced?

Question 8: Do you think gender play a role when families make decisions?
Probing:
- Is it different if it is a boy or a girl that falls sick? Why?

Question 9: Services for children are supposed to be provided for free, but many don't seek care. How have you seen that high costs influence the families' decisions to seek care?
Probing:
- How have you experienced this in your practice?
- How do other factors play a role? Do you know of an example…

Question 10: How does illness of the newborns influence the rest of the family?
Probing
- What are the benefits for the family? Baby survives?
- What are the burdens? Less spending on food? Less spending on education? Experiences of sales (of belongings), spending of savings, borrowing from others?
- Can you give an example from your practice?

Question 11: How are the different family members involved in decision making [to seek care/stay at home/go to traditional practitioner]?
Probing:
- Mother, father, older siblings, mother in law, grandmothers, others?
- If there is disagreement, what is it about? How do you think is agreement made?
- Who do you think has the final say? Why?
- How have you seen these processes been dealt with in the ward?

I have now asked you questions about ill newborns and how families make decisions about health care seeking. I have some general questions which you might have some thoughts on:

Question 12: Some studies from West Africa and Asia show that newborns are valued of less importance than adults: How is a newborn death perceived versus an adult death in this community?
Probing:
- How is a newborn death perceived versus a death of an older child?
- Why do you think it is like this?

Question 13: What do you think of the government's policies to address newborn health?
Probing:
- What must be done to give newborns a higher status? How can this be done?

Question 14: Do you have anything you would like to add to what we have talked about?

Thank you for your participation and time.

Topic guide – focus group discussions: Health care workers

Site:

Date and time:

Name of moderator:

Introduction

Welcome to this group discussion, and thank you for agreeing to speak with us today. My name is ___________ and my colleague's name is ________. We are researchers at the School of Public Health, Addis Ababa University, Ethiopia and the Department of Global Public Health, University of Bergen, Norway. We are doing a study to learn more about care of sick newborns and the use of health care services. We also seek to understand the social and economic consequences for families with sick babies. We hope that this study will give important knowledge for health care services to newborns.

We would like to conduct a group discussion today with you as health professionals with experience in caring for sick newborns.

Procedure

We are interested in your thoughts and experiences, and there are no right or wrong answers. We would like to hear all points of view and encourage you to feel free to disagree with one another. All comments are welcome. To allow good discussions, we would ask you to not discuss details of this conversation after you leave this room. We also would like to encourage you to speak one at a time.

Before we begin, I would like to read aloud the informed consent.

[Interviewer reads the informed consent and asks the participants for questions. The participants will sign the form or withdraw from the study]

As I mentioned [in the informed consent], I would like to use a tape recorder to record our group discussion. I would like to emphasise again that the information you provided will be confidential. Any information we use from this group discussion will be combined with other information and it will not be possible to identify what you said.

Do you have any questions before we start?

Question 1: We will now discuss difficult decisions the family might have to make when a baby is ill.
Please tell us about a baby that was taken to your hospital after falling sick.
Probing:

- What happened to the baby?
- What happened to the family?

Question 3: What do you think the family consider when deciding to take a baby to the hospital/health center?
Probing:

- What do you think is important for the family members in their considerations (the family economy, the baby, the father's mother)?

- Why would go to the hospital?

Question 4: How would you as a health care professional respond if the family decides to leave the hospital?
Probing:
- What would you do if you think the family is about to leave the hospital against your advice?
    - Do you play a role in protecting the interests of the baby? Who plays a role? The other family members?

Question 5: Have you experienced that a newborn has been left in the hospital or a baby was brought to hospital without any parents or family? What did you do?
- Why do you think they left the baby?
- Who are responsible for taking care of these babies?
- How did you understand that they were leaving the hospital?

We will now discuss difficult decisions the family might have to make when a newborn is ill.
Question 6: What concerns are most important to the family if a newborn fall ill?
- Is the newborn a priority? Why/why not?
- Can you give an example from your clinical work?
- What if one of the older brothers, the father or the grandmother (father's mother) also has fallen sick. If they have to choose, should they treat the baby or the grandmother?

Question 7: Do you think gender of the newborn play a role when families make decisions?
Probing:
- Would it be different if it was a boy that fell sick? Why?

Question 8: Services for children are supposed to be provided for free, but we many don't seek care. How do you think high costs influence the families' decisions to seek care?
Probing:
- How have you experienced this in your practice?
- How are other factors important to health care seeking? Do you know of an example…

Question 9: What are the consequences for the families' of using their resources on ill babies?
Probing:
- What are the benefits for the family? Baby survives?
- What are the burdens? Less spending on food? Less spending on education? Experiences of sales (of belongings), spending of savings, borrowing from others?
- How have you experienced that in your practice?

Question 10 Some studies from West Africa and Asia show that newborns are valued of less importance than adults: How is a newborn death perceived versus an adult death in this community?
Probing:
- How is a newborn death perceived versus a death of an older child?
- Why do you think it is like this?

Question 11: What do you think of the government's policies to address newborn health?
Probing:
- What must be done to give newborns a higher status? How can this be done?

Question 12: Do you have anything you would like to add to what we have talked about?

Thank you for your participation. Your time is very much appreciated and your comments have been very helpful.

Topic guide – focus group discussions: Community members

Site:

Date and time:

Name of moderator:

Introduction

Welcome to this group discussion, and thank you for agreeing to speak with us today. My name is ___________ and my colleague's name is ________. We are researchers at the School of Public Health, Addis Ababa University, Ethiopia and the Department of Global Public Health, University of Bergen, Norway. We are doing a study to learn more about care of sick babies and the use of health care services. We hope that this study will give important knowledge to improve health care services to babies.

Procedure

We are interested in your thoughts and experiences, and there are no right or wrong answers. We would like to hear all points of view and encourage you to feel free to disagree with one another. All comments are welcome. To allow good discussions, we would ask you to not discuss details of this conversation after you leave this room. We also would like to encourage you to speak one at a time.

Before we begin, I would like to read aloud the informed consent.

[Interviewer reads the informed consent and asks the participants for questions. The participants will sign the form or withdraw from the study]

As I mentioned [in the informed consent], I would like to use a tape recorder to record our group discussion. I would like to emphasise again that the information you provided will be confidential. Any information we use from this group discussion will be combined with other information and it will not be possible to identify what you said.

Do you have any questions before we start?

Question 1: What do you think the family would do in this case?
Probing:
- Why would they respond in this way?
- What do you think is the family's opinion about the advice from the doctor?
- What are important for them (the family economy, the baby, the father's mother)?

We will now discuss difficult decisions the family might have to make when a baby is ill.
Question 1: Please tell us about your experiences when a baby has fallen sick. Probing:
- What happened to the baby?
- What happened to the family??
- Can you give me an example?

Question 2: What kind of difficult decisions do families in this community face when babies fall ill (like in the cases we just discussed)?

- Which concerns are (most) important to the family?

Question 3: In the cases you described, imagine that it is not only the baby that is sick. What if other family members become ill? Is it more important to care for the baby or the other family members?
Probing:
- Imagine that one of the older brothers also has fallen sick. If they have to choose, should they treat the baby or the brother? Why?
- Imagine that the mother also has fallen sick. If they have to choose, should they treat the baby or the mother? Why?
- Imagine that the father also has fallen sick. If they have to choose, should they treat the baby or the father? Why?
- Imagine that the grandmother (father's mother) also has fallen sick. If they have to choose, should they treat the baby or the grandmother? Why?

Question 4: How does the gender of a baby play a role?
Probing:
- Would it be different if it was a boy or a girl that fell sick? Why?

Question 5: Services for children are supposed to be provided for free, but many don't seek care. What are the costs you experience when going to the hospital or health facilities?
Probing:
- How do you think high costs influence the families' decisions to seek care for babies?
- How do you deal with the high costs?
- Do you know of an example…

Question 6: What are the consequences for the families' of using their resources on ill babies?
Probing:
- *What are the benefits for the family?* Baby survives?
- *What are the burdens?* Less spending on food? Less spending on education? Experiences of sales (of belongings), spending of savings, borrowing from others?

Question 7: What should the family choose, if the family has to sell a cow to take the baby to a facility?
Probing:
- What are most important if other family members also are in need of resources?
- How do think this is dealt with in practice?

Question 8: Some say that newborns are less important than adults: How is a newborn death perceived versus an adult death in your community?
Probing:
- Or a death of a newborn versus a death of an older child?
- How does your opinion differ from this? Do you agree with what he/she said?
- Why do you think newborns are perceived of lower/higher importance?

Question 9: Do you have anything to add that you we have talked about?

Thank you for your participation. Your time is very much appreciated and your comments have been very helpful.
